# Sweet lies: neural, visual, and behavioral measures reveal a lack of self-control conflict during food choice in weight-concerned women

**DOI:** 10.3389/fnbeh.2014.00184

**Published:** 2014-05-22

**Authors:** Laura N. van der Laan, Denise T. D. de Ridder, Lisette Charbonnier, Max A. Viergever, Paul A. M. Smeets

**Affiliations:** ^1^Image Sciences Institute, University Medical Center UtrechtUtrecht, Netherlands; ^2^Department of Clinical and Health Psychology, Utrecht UniversityUtrecht, Netherlands; ^3^Division of Human Nutrition, Wageningen University and Research CentreWageningen, Netherlands

**Keywords:** decision-making, food choice, visual attention, conflict monitoring, fMRI

## Abstract

Despite their intentions, weight-concerned individuals generally fail to control their eating behavior. However, it is unknown whether this failure is due to a lack of effortful self-control, or to not experiencing an internal conflict between weight goals and food temptations. The present study used fMRI, eye tracking and reaction times to assess the degree of conflict experienced by weight-concerned women during food choices that posed either a self-control dilemma (i.e., requiring a choice between healthy and palatable foods), or not. Contrary to the common assumption in self-control theory that food choices posing a self-control dilemma evoke internal conflict, we found that choices requiring self-control induced no conflict, as demonstrated by lower reaction times, fixation durations, number of gaze switches between snacks, and lower activation of the anterior cingulate cortex. Our results suggest that self-control failure might be due to a lack of experienced conflict, rather than to failing to act upon the perception of such conflict. This implies that effectiveness of weight maintenance interventions might be improved if they also focus on increasing the ability to detect a self-control dilemma, in addition to the current focus on increasing self-regulatory capacity.

## Introduction

Approximately 50–60% of the Western female population guard themselves against the risks of the obesogenic environment by attempting to limit their energy intake (Rideout and Barr, 2009; Fayet et al., 2012; de Ridder et al., in press). However, despite their intentions, weight-concerned individuals generally fail to control their food intake: they do not eat less than non-weight-concerned counterparts (Stice et al., 2004, 2007, 2010; de Witt Huberts et al., 2013) and their ratings of weight concerns even predict future weight gain (French et al., 1994; Mann et al., 2007).

The absence of a relation between self-reports of weight-consciousness and food intake indicates that high scores on restraint and weight-concerns are a measure of intention rather than a predictor of behavior (e.g., Stice et al., 2010). For weight-concerned individuals, choosing between healthy and (more tasty) unhealthy foods is regarded as a classic self-control dilemma involving the trade-off between immediate eating enjoyment and the future benefits of being slim and healthy (Fishbach et al., 2003). In order to resolve this dilemma and behave in line with their intentions, self-control should be exercised (Baumeister and Heatherton, 1996; Fishbach et al., 2003). Examples of such self-control strategies are bolstering the value of the long-term goal or using other cognitive strategies aimed at keeping the long-term goal in the focus of attention (e.g., Metcalfe and Mischel, 1999; Peake et al., 2002).

At the heart of self-regulation theory lies the assumption that if someone with the long-term goal to limit intake is faced with a food choice that threatens the accomplishment of this goal, this results in the experience of an internal conflict (Fishbach et al., 2003). Consequently, self-regulatory failure is usually attributed to the subsequent inability to resolve the conflict in favor of the long term goal. This has led to a large number of studies aimed at improving individuals' ability to overcome the self-control dilemma [e.g., by cognitive strategies, (Siep et al., 2012; Giuliani et al., 2013)]. However, the assumption that a self-control dilemma evokes internal conflict has so far been untested and therefore it remains unknown whether self-regulatory failure is due to a lack of cognitive control, or to the absence of experienced conflict. This topic is of major relevance for interventions aimed at weight loss or maintenance since it will elucidate whether interventions should aim only at strengthening self-regulatory capacity and cognitive control or also at strengthening the ability to detect a self-control conflict.

Conflict monitoring is the process by which the brain determines when control is required (Botvinick et al., 2001) and thus precedes the actual act of self-control (exerting cognitive control). Although there is a large body of research on self-control and the neural correlates of self-control in food choice (Hare et al., 2009, 2011; Heatherton and Wagner, 2011; de Ridder et al., 2012), studies specifically assessing conflict monitoring during food choice in weight-concerned women are lacking.

In response to this, the study aim was to investigate the levels of conflict experienced by weight-concerned women during food choices that posed either a self-control dilemma or not. As the perception of conflict is not necessarily a conscious phenomenon, we used measures that assess implicit cognitive (reaction times during food choice, reaction times in a lexical decision task), attentional (eye tracking) and neural processes [functional Magnetic Resonance Imaging (fMRI)]. Previous studies have demonstrated that response conflict in a task (i.e., task difficulty) is accompanied by higher reaction times (e.g., Panayiotou and Vrana, 2004), more gaze switches between items (e.g., Causse et al., 2011), and stronger activation of the anterior cingulate cortex (Botvinick et al., 2001; van Veen et al., 2001). Implicit measures are less susceptible to demand characteristics and socially desirable responding (Fazio and Olson, 2003). This is especially relevant for weight-concerned women who might be inclined to respond in line with their intention to limit their food intake rather than their actual behavior.

## Methods

### Ethics statement

The study was approved by the Medical Ethical Committee of the University Medical Center Utrecht and participants provided written informed consent.

### Participants

The study comprised of 20 women as participants (age in years: *M* = 21.2, *SD* = 2.8; BMI in kg/m^2^: *M* = 21.3, *SD* = 1.7). Participant selection was limited to women because they generally score higher on weight concerns and because of known gender differences as well in reasons for dieting as in brain anatomy and function (Pingitore et al., 1997; Neumark-Sztainer et al., 1999; Cahill, 2006; Luders et al., 2009). Earlier studies have shown that there are gender differences in brain responses to food cues (Smeets et al., 2006; Cornier et al., 2010; Frank et al., 2010; Haase et al., 2011). As self-control conflict is only relevant for individuals who are weight-concerned, inclusion criteria consisted of a restraint-score above average or high (Dutch Eating behavior questionnaire Van Strien et al., 1986) and a rating of 6 or higher on each of two questions: “To what extent are you weight-concerned?” and “To what extent are you occupied with being slim?” (ranging from 1 = not at all to 9 = very much; adapted from Fishbach et al., 2003). Additional inclusion criteria were having an age between 18 and 30 years and having a normal weight (BMI between 18.5 and 25 kg/m^2^).

Being left-handed was an exclusion criterion because the brains of left and right-handed individuals are generally thought to differ in anatomical and functional characteristics (Guadalupe et al., 2014; Ooki, 2014). By only including right-handed individuals (right-handed because the majority of the population is right-handed), we aimed to reduce the possible variation in functional responses in our study population. Further exclusion criteria consisted of having a food allergy, having an eating disorder, and having a history of medical or surgical events that might significantly affect the study outcome, such as metabolic or endocrine disease, or any gastro-intestinal disorder. Smokers and individuals having a current alcohol consumption of >28 units per week were excluded because these factors have been shown to affect the neural response to rewarding stimuli. We excluded women that followed a medically prescribed diet in the past 6 months or that had weight fluctuations of more than five kg in the past six months so as to exclude participants who may show biases in their food choices for medical reasons and as extra caution to exclude individuals with eating disorders or problematic eating behavior, who might have aberrant brain responses to food and thereby introduce heterogeneity in the sample. Participants were recruited with posters at the University Medical Center Utrecht and the adjacent university campus.

### Procedures

The study consisted of two sessions (on two separate days with 1–8 days in between). During the first session, participants completed several questionnaires and computer tasks, including a primed lexical decision task (see Lexical decision task). Moreover, participants evaluated the expected tastiness and perceived energy content of the food stimuli on 9-point scales ranging from 1 = very untasty/very few calories to 9 = very tasty/very many calories. No imaging data was collected during this first session.

In addition to this, the complete test-battery of questionnaires and tasks included: the Behavioral Avoidance/Inhibition (BIS/BAS) Scales (Carver and White, 1994), the Health and Taste Attitudes Scales (Roininen et al., 2001), the Barrats Impulsiveness Scale (Patton et al., 1995), the Brief Self-Control Scale (Tangney et al., 2004), the N-Back task (Kirchner, 1958), the traditional STROOP task (Stroop, 1935), and the Kirby Delay Discounting task (Kirby et al., 1999). Results from these measures are not reported here.

To make sure participants were craving for a snack, they were instructed to refrain from eating and drinking (except water) for at least 2 h prior to the second session but to have preferably eaten a meal within 2–3 h before the session (mean time since last food intake in minutes ± *SD*: 140 ± 22). Upon arrival, participants received instructions and rated hunger on a VAS scale ranging from 0 (not hungry) to 100 (very hungry) (mean hunger ± *SD*: 59 ± 12). Subsequently, to ensure the relevance of their weight-concerns participants filled out a questionnaire about a pro-claimed new type of snack-biscuit (questions about expected tastiness, expected energy content and to what extent eating the biscuit is appropriate for individuals who are watching their weight). Thereafter, participants were scanned with a 3 Tesla Philips Achieva scanner (Philips Healthcare, Best, Netherlands), while performing a food choice fMRI task. During this task, eye movements were recorded using an MR-compatible eye-tracker (Nordic Neurolabs, 60 Hz). Besides the food choice task reported here, participants also performed another, unrelated, food choice task. Finally, participants received one of the snacks they chose in the food choice task, were thanked and reimbursed.

### Lexical decision task

**Figure 1:**
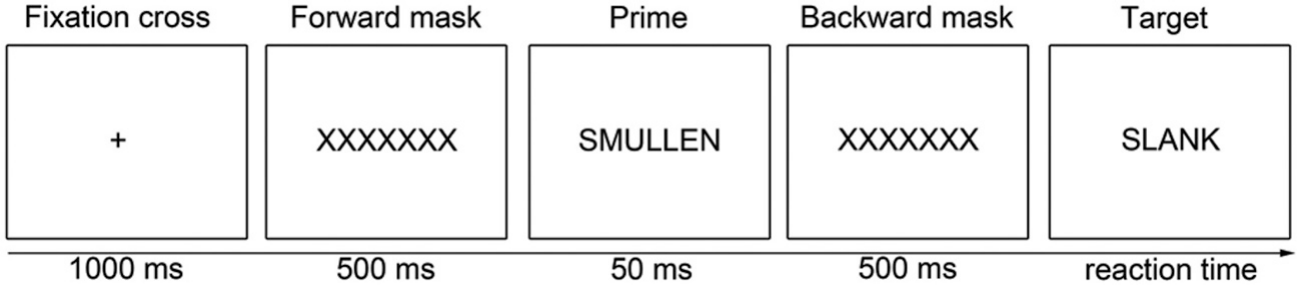
**Task structure of the primed lexical decision task measuring temptation-goal associations**. “Smullen” is a temptation-prime (Dutch for “feasting”). “Slank” is a diet-target (Dutch for “slim”).

Since temptation-goal associations have been shown to mediate successful self-control, a primed lexical decision task (Figure 1) with temptation/neutral primes and diet/neutral target words was used to measure the strength of temptation-goal associations (adapted from Kroese et al., 2011).

The lexical decision task consisted of 144 trials comprising a fixation cross (1000 ms), a forward mask (“XXXXXX”; 500 ms), a prime word (50 ms), a backward mask (“XXXXXX”; 500 ms), and a target letter string. The target letter string stayed on the screen until participants pressed the z or m button of the keyboard to indicate that the target was a word or a non-word, respectively. Half of the 144 targets were non-word targets (12 non-words, each repeated 6 times), 54 were neutral targets (9 neutral words, e.g., bell, purple, finding, each repeated six times), and 18 were diet targets (3 diet related words, i.e., dieting, slim, thin, each repeated six times). Three temptation primes were used [i.e., chocolate, feasting (“smullen”) and eating candies (“snoepen”), each repeated 6 times] and 21 neutral primes (e.g., letter, contact, normal, each repeated six times). The temptation and neutral primes were matched on word length, as were the diet-related and neutral targets.

The primes and targets were combined such that four trial categories of interest were created: (1) a neutral prime with a diet target (nine trials), (2) a temptation prime with a neutral target (nine trials), (3) a temptation prime with a diet target (nine trials), and (4) a neutral prime with a neutral target (45 trials).

For responses in the lexical decision task, (log) reaction times were aggregated over participants, prime (neutral or temptation) and target type (diet target or neutral target). A two-level (trial types nested within participants) regression model was performed with prime type, target type and the effect of interest: the interaction between prime and target.

### fMRI food choice task

**Figure 2:**
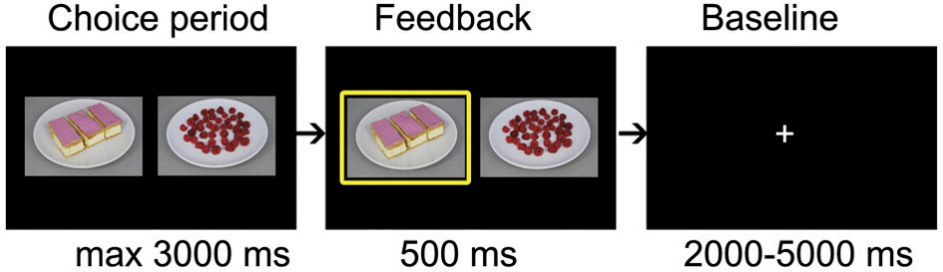
**fMRI food choice task structure**.

In the food choice task (Figure 2), participants made a total of 100 choices. In every trial, a high energy (HE) (energy content in kcal/100 gram: *M* = 419, *SD* = 103) and a low energy (LE) (*M* = 56, *SD* = 37) snack were shown side by side. Participants had 3000 ms to indicate which of the two products they would most like to eat a portion of by pushing the left or right button of a button box. After indicating their choice, a yellow box appeared around the chosen product for 500 ms. The trials were interspersed with a random interval between 2000 and 5000 ms.

To investigate response conflict during food choice participants were required to choose between pairs of HE and LE snacks matched such (on the basis of their own tastiness ratings given in the first session) that either a self-control dilemma was posed or not. In half of the trials, LE snacks were combined with HE snacks rated two or three points higher on tastiness [Self-Control required (SC) trials], posing a self-control dilemma concerning the trade-off between immediate eating enjoyment (choosing the appealing HE snack) and weight watching intentions (choosing the less appealing LE snack). In the other half of the trials, the LE and HE snacks were matched on tastiness [equal or ±1 point in tastiness rating; No Self-Control required (NSC) trials], such that no trade-off between eating enjoyment and weight watching intentions was needed to choose the long-term superior LE snack. Participants were not told explicitly that the choices were always between pairs of high and low energy snacks.

To avoid that participants had to choose between products that they highly disliked, only stimuli with a tastiness rating of four or higher were used. For two participants, the tastiness ratings allowed the construction of only 40 SC trials (instead of 50), without repeating stimuli more than 10 times. Successful SC trials were defined as those in which the LE snack was chosen and unsuccessful SC trials as those in which the HE snack was chosen.

### Eye tracking data analysis

Fixation detection was established by marking fixations with an adaptive velocity threshold method (Arrington Research) with the default lower cut-off for fixation duration of 75 ms. In order to analyze fixation behavior, the screen was divided in three regions, namely: left product, the left 42% of the screen; right product, the right 42% of the screen and middle, the middle 16% of the screen.

A fixation was considered last fixation if it was the final fixation on a product preceding the button press indicating the participant's choice. Total fixation duration was defined as the sum of the durations of all fixations on a product in a trial.

For four participants no stable eye tracking data could be acquired. These participants were removed from the analyses concerning eye tracking data.

### Behavioral data analysis

In the food choice task stimuli were nested within trials, and trials were nested within participants. Therefore, a series of multi-level regression analyses was performed to investigate how visual measures relate to choice. For outcomes on the stimulus level (the stimulus being chosen or not, total fixation duration on HE/LE snacks and last fixation) three-level regression analyses were performed, and for outcomes on the trial level (reaction times and total fixation duration for HE and LE snack summed) two-level regression analyses were performed. For continuous outcome variables (fixation duration and reaction times) linear regression analyses were performed. For binary outcome variables (the stimulus being chosen or not) logistic regression analyses were performed.

To correct for a non-normal distribution, natural log-transformed reaction times were used in all analyses. Extreme reaction times [>3 *SD* from the (log- transformed) mean] were set to missing.

The statistical program R (packages lme4 and languageR) was used to perform multi-level regression analyses.

### fMRI data

#### Image acquisition and preprocessing

MRI scanning was performed on a 3 Tesla scanner (Philips Achieva, Philips Health- care, Best, Netherlands), equipped with a SENSE head coil. A T1 -weighted structural image was acquired at a resolution of 1 × 1 × 1 mm (*TR* = 8.4 ms, *TE* = 3.8 ms, total scan duration = 284 s). Functional scans were acquired with a 2D-EPI sequence (*TE* = 23 ms, flip angle = 70°, nr slices = 30, voxel size = 4 × 4 × 4 mm, acquisition time of one 3D volume = 1400 ms). The total number of volumes acquired differed between participants because of the random inter-trial interval. Data were preprocessed and analyzed using the SPM8 software package (Wellcome Department of Imaging Neuroscience, London, United Kingdom) ran with MATLAB R2012A (The Mathworks Inc, Natick, MA). Functional images were realigned to the first image of the time series. Functional and structural images were co-registered and normalized (retaining 4 × 4 × 4 mm voxels) to MNI space (Evans et al., 1993) by using linear and non-linear transformations.

#### Participant level analysis

Statistical maps were generated for each participant by fitting a boxcar function to the time series, convolved with the canonical hemodynamic response function. Data were high-pass filtered with a cutoff of 128 s. Two conditions were modeled: the choice periods of the SC trials and the choice periods of the NSC trials. For each participant, two contrast images were calculated: (1) to establish the brain regions that responded more strongly during SC food choices, we performed a mean subtraction analysis between SC and NSC trials, (2) to establish the brain regions that respond stronger during NSC food choices, we performed a mean subtraction analysis between NSC and SC trials.

#### Group level analysis

The anterior cingulate is regarded as a primary region in conflict monitoring (Botvinick et al., 2001; van Veen et al., 2001). Therefore the left and right anterior cingulate were our a priori Regions Of Interest (ROIs). The ROI masks were generated using the AAL-atlas (Tzourio-Mazoyer et al., 2002) as implemented in the WFU-pickatlas toolbox (Maldjian et al., 2003). To determine whether the anterior cingulate shows differential activation for SC and NSC trials, the contrast images were entered into a one-sample *t*-test. For the anterior cingulate ROIs a statistical threshold of *p* < 0.05 Family Wise Error (FWE)-corrected over the ROI volume (i.e., small volume correction) was used.

For completeness and to enable future meta-analysis, we also report whole brain results at a statistical threshold of *p* < 0.005 uncorrected and a cluster extent *k* ≥ 10 voxels, in Supplementary Table S1.

## Results

### Choice behavior

In the NSC condition, 45.9% HE choices were made and 54.1% were LE choices. In the SC condition, 78.9% were HE choices and 21.1% were LE choices.

### Reaction time during food choice

**Figure 3:**
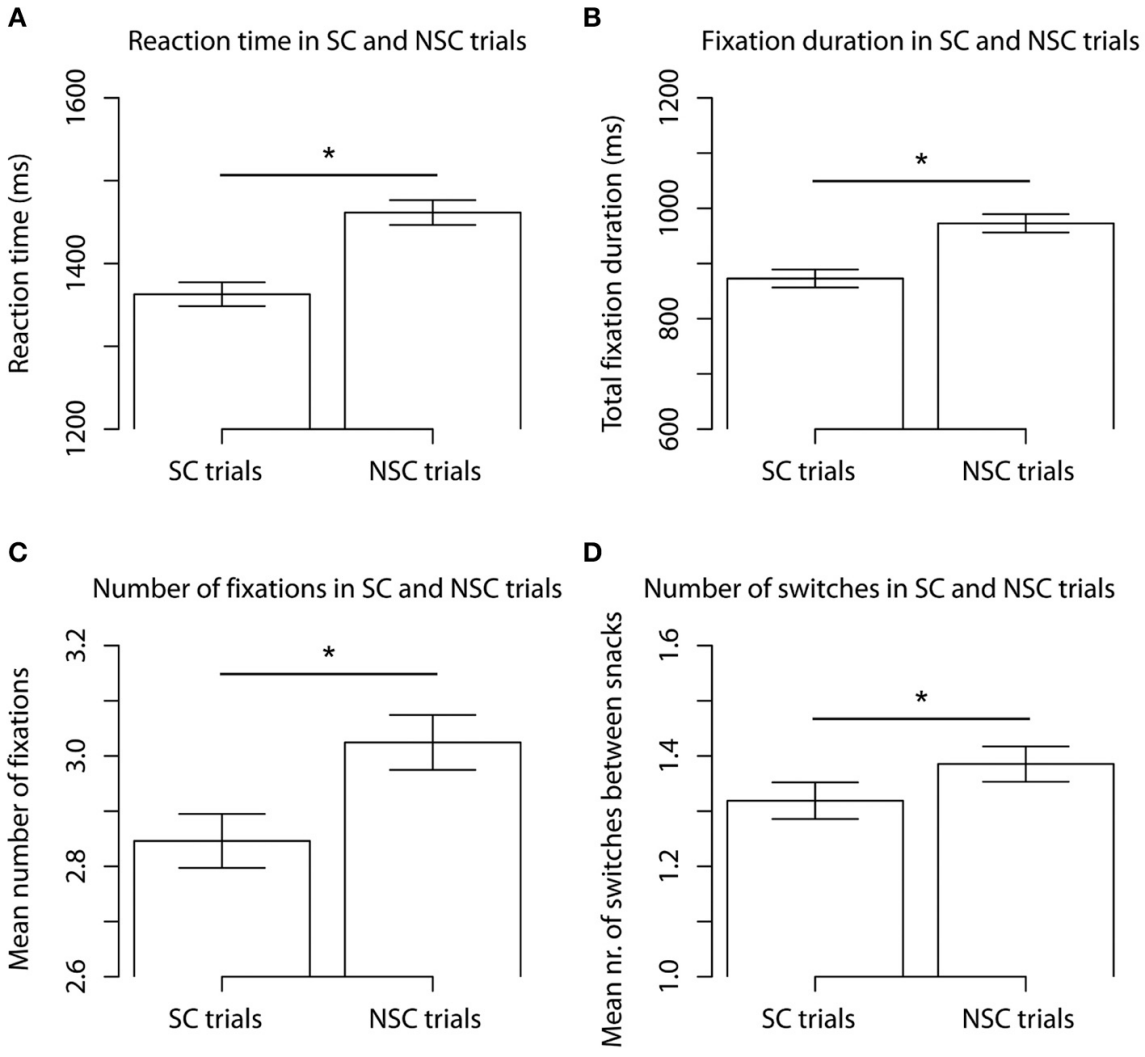
**Mean reaction times (A), total fixation duration on HE and LE snack summed (B), number of fixations on HE and LE snack summed (C) and number of switches between HE and LE snacks (D), for SC and NSC trials**. Barplots show mean ± s.e.m. ^*^p < 0.05.

**Figure 4:**
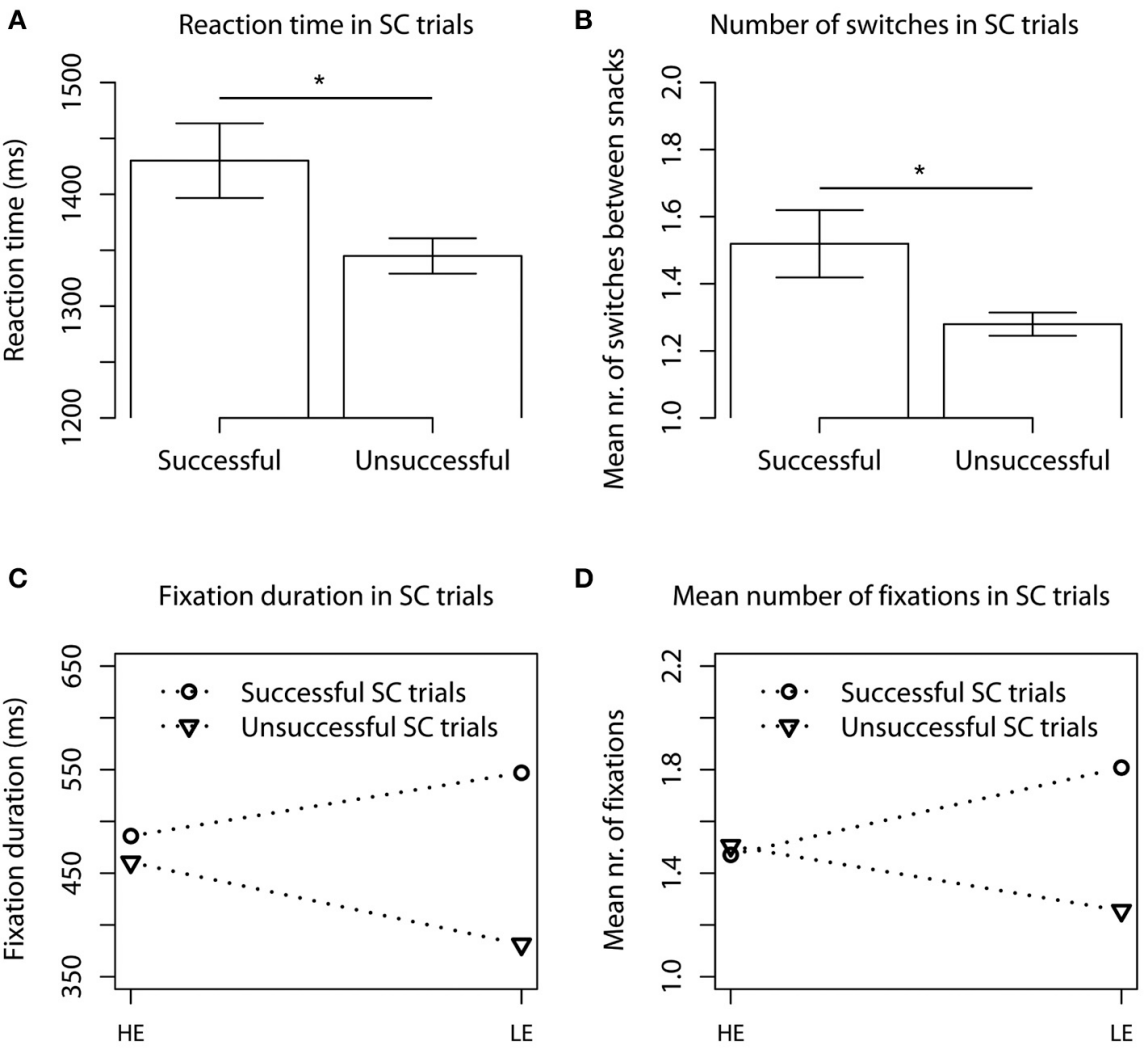
**Mean reaction times (A) and number of gaze switches between snacks (B) in successful and unsuccessful SC trials**. Barplots show mean ± s.e.m. Mean total fixation duration **(C)** and number of fixations **(D)** on HE and LE snacks in successful and unsuccessful SC trials. ^*^p < 0.05.

Mean reaction time during the food choice task was 1412 ms (*SD* = 452 ms). Regression analysis showed that reaction time was significantly higher for NSC (*M* = 1462 ms, *SD* = 460 ms) compared to SC trials (*M* = 1363 ms, *SD* = 439 ms, *p* < 0.01, Figure 3A, Table S2). The regression analysis revealed that in the successful SC trials reaction times (*M* = 1430 ms, *SD* = 468 ms) were significantly higher compared to unsuccessful SC trials (*M* = 1345 ms, *SD* = 429 ms, *p* < 0.01, Figure 4A).

### Total fixation duration and number of fixations

The regression analysis showed that total fixation duration (sum of fixation durations on left and right product summed) was higher in the NSC (*M* = 973 ms, *SD* = 450 ms) compared to the SC trials (*M* = 873 ms, *SD* = 437 ms, *p* < 0.01, Figure 3B, Table S3). The same pattern was found for the number of fixations (Figure 3C).

A regression analysis explaining fixation duration in SC trials with factors energy content of product fixated on (HE or LE), and success (i.e., successful if LE is chosen and unsuccessful if HE is chosen), and an interaction term of energy content × success revealed a significant interaction (*p* < 0.01) between energy content and success. That is, in successful SC trials LE stimuli were fixated on longer than in unsuccessful SC trials. This interaction effect is plotted in Figure 4C. The same pattern was found for the number of fixations (Figure 4D).

### Number of switches between items

The regression analysis showed that the number of gaze switches between snacks was higher in the NSC (*M* = 1.4, *SD* = 0.9) than the SC condition (*M* = 1.3, *SD* = 0.9, *p* < 0.05, Figure 3D). In successful SC trials (*M* = 1.5, *SD* = 1.0) participants switched their gaze significantly more often between snacks than in unsuccessful SC trials (*M* = 1.3, *SD* = 0.9, *p* < 0.01, Figure 4B).

### Location of last fixation

The last fixation was on a HE stimulus in 50.3% of the trials. To investigate whether visual patterns differed between successful and unsuccessful SC trials, we conducted a regression analysis explaining last fixation location in SC trials with factors energy content of product fixated on last (HE or LE), and success (i.e., successful if LE is chosen and unsuccessful if HE is chosen) and an interaction term of energy content × success. This analysis revealed a significant interaction (*p* < 0.01) between energy content of last product fixated on and success. That is, in successful SC trials, the last fixation was in 70.2% of the trials on the LE snack (29.8% on HE snack); in unsuccessful SC trials, the last fixation was in 40.5% of the trials on the LE snack (59.5% on HE).

### Temptation-goal associations

Reaction times in the lexical decision task were used to establish temptation-goal associations. If the prime activates the weight-watching goal, the reaction to diet targets preceded by a temptation prime would be quicker than the reaction to diet targets preceded by a neutral prime. Therefore, we conducted a linear regression (trial type nested within participants) on reaction time with target (diet or neutral), prime (temptation or neutral) and the interaction term of target × prime. This analysis showed that neither the effect of interest (the interaction between target and prime; *p* = 0.83), nor the main effects of target (*p* = 0.64) and prime (*p* = 0.63) were significant (Table S4).

### fMRI results SC vs. NSC trials

**Figure 5:**
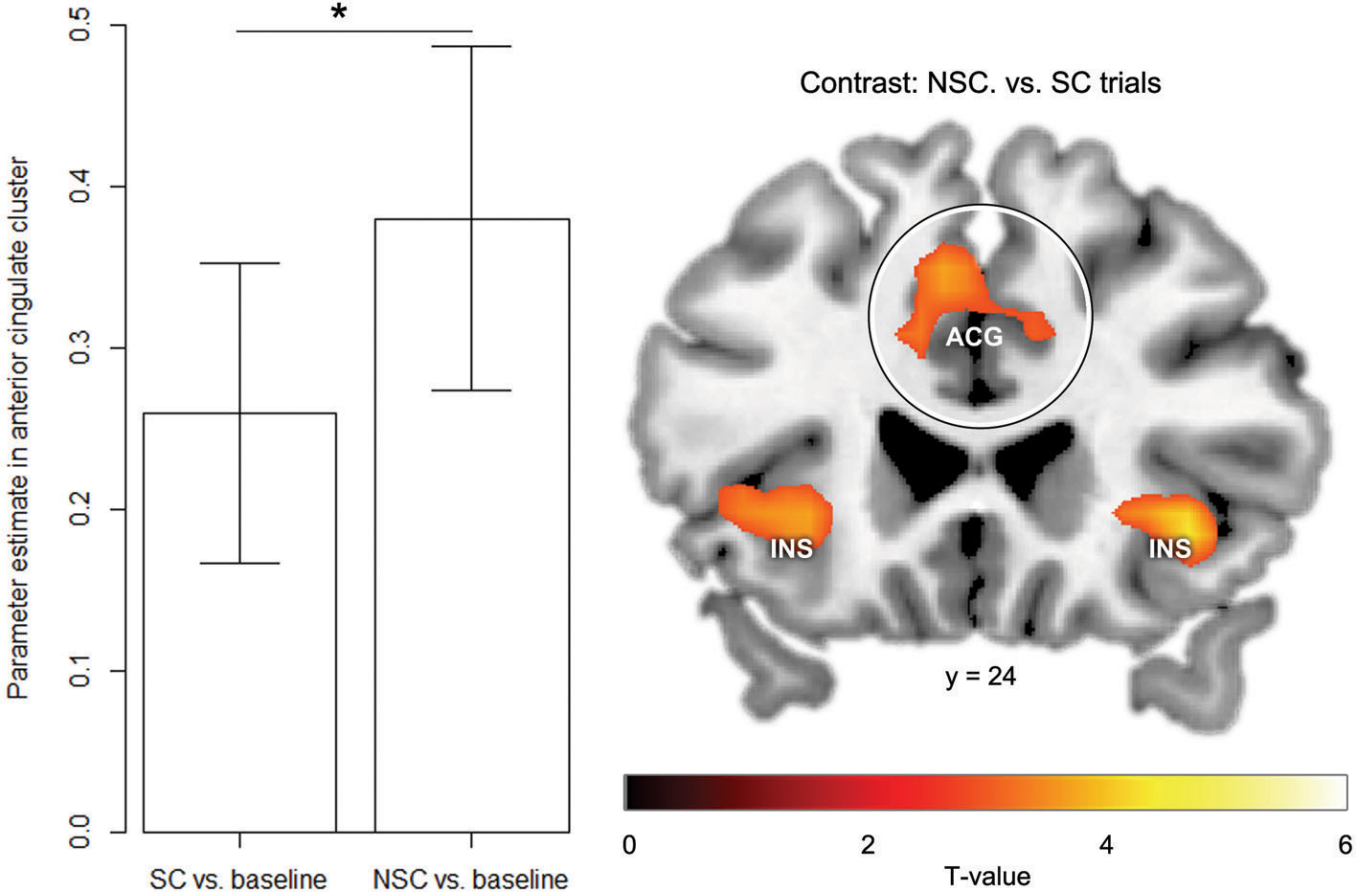
**fMRI results reveal stronger anterior cingulate activation in NSC vs. SC trials**. Left panel: Mean ± s.e.m. parameter estimates in anterior cingulate cluster for both conditions vs. baseline. Right panel: fMRI results for contrast NSC vs. SC trials. Circle indicates anterior cingulate cluster. For visualization purposes, fMRI-results are thresholded at *T* > 2.87, *p* < 0.005 uncorrected. ACG, Anterior cingulate cluster; INS, Insula. ^*^p < 0.05.

The subtraction analysis of NSC vs. SC trials revealed that a cluster in the left anterior cingulate [*p* = 0.017 FWE-corrected for ROI volume, *Z* = 3.51, peak coordinate MNI (−10, 28, 26)] was stronger activated during NSC compared to SC trials (Figure 5). Results from whole-brain analysis are reported in Table S1.

## Discussion

Our aim was to investigate levels of experienced conflict during food choices made by weight concerned women. In accordance with previous studies (Stice et al., 2004, 2007, 2010), we found that participants were generally unsuccessful in choosing in line with their long term weight-watching goal: the LE snack was chosen in only 21.2% of the trials posing a self-control dilemma (choosing between a LE and a more tasty HE snack, SC trials) and in 54.1% of the trials that did not pose a self-control dilemma (choosing between equally liked HE and LE snacks, NSC trials). Our results showed that during the choices that posed no self-control dilemma the reaction times, fixation duration and number of gaze switches were higher compared to trials that did pose a self-control dilemma. It is well established that reaction times, fixation durations and number of gaze switches are higher in difficult tasks requiring the recruitment of cognitive resources (Panayiotou and Vrana, 2004; Causse et al., 2011). Furthermore, a cluster in the anterior cingulate cortex, which has previously been shown to activate during the perception of conflict (Botvinick et al., 2001; van Veen et al., 2001), was more strongly activated during trials that did not pose a self-control dilemma (NSC trials).

Hence, in sharp contrast with psychological theories on self-control, the behavioral, eye tracking and neural findings suggest that a stronger response conflict was experienced during the trials in which no self-control dilemma was posed (NSC trials). For weight-concerned individuals the NSC choices should have constituted an easy choice that requires no self-control because the LE snack was as tasty as the HE snack while being in line with their weight-watching goal. In contrast, the SC trials should have constituted a difficult choice because the preferred HE snack is not conducive to their weight-watching goal and thus self-control was required to choose the LE snack over the tastier HE snack. Our findings suggest that weight-concerned women do not experience difficulty or internal conflict during choices posing a self-control dilemma and that this leads to food choices that are primarily guided by tastiness and not by energy content. This supports the notion that self-regulatory failure might be due to a lack of experienced internal conflict rather than a failure to act upon the perception of such conflict.

Having established that self-regulatory failure might be due to a lack of internal conflict, the question arises why self-proclaimed weight-concerned women do not appear to experience internal conflict when confronted with a self-control dilemma. A possible explanation might be that exposure to the appealing HE snack decreased the accessibility of their long term goal to watch their weight. This mechanism, concerning the facilitating role of temptations on indulgence, has been provided by the goal-conflict model (Stroebe et al., 2008), which posits that exposure to temptation inhibits the accessibility of the opposing long-term goal. However, there is a contrasting line of research based on counteractive-control theory (Trope and Fishbach, 2000) that posits that temptations may assist, rather than undermine, long-term goal attainment (Kroese et al., 2011; Smeets et al., 2013). That is, temptation cues activate rather than inhibit long-term goal accessibility. To explore how exposure to tempting HE foods might have influenced the accessibility of the weight-watching goal, we used a primed lexical decision task to measure temptation-goal associations. This task revealed that exposure to temptation cues did not appear to activate the long term weight- watching goal. This is in line with an earlier study that showed that temptation cues activate long term goals only in successful, and not in unsuccessful self-controllers (Papies et al., 2008). Thus, our results suggest that the lack of experienced conflict might be explained by the absence of temptation-goal associations, implying that exposure to appealing HE snacks within binary choice sets requiring self-control does not increase the accessibility of the weight-watching goal. This could be further tested in future studies that measure the accessibility of the weight-watching goal directly in response to different choice pairs either requiring self-control or not, such as the choices that we used in our study. For example, reaction times to dieting and neutral words, after a prime choice-set that either requires self-control or not, could be measured. Most current studies on temptation-goal associations assess goal accessibility by measuring reaction times to diet and neutral words after primes with single words or pictures of temptation stimuli. Investigating the accessibility of the weight-watching goal in response to different choice sets varying in aspects like the need for self-control, is an important direction for future research.

From theory it follows that having a long-term goal is a prerequisite for perceiving an internal conflict in response to a self-control dilemma (e.g., Fishbach et al., 2003). For this reason, we only included participants that were weight-concerned, according to self-reports. However, the finding that our participants did not experience conflict questions whether they are truly dedicated to restrict their energy intake. Interesting to note is that we found exactly what would be expected for individuals who are not weight-concerned (i.e., people without the goal to limit their energy intake). For non-weight-concerned individuals, tastiness is the main predictor of choice, while the energy content of the food does not play a role (Arvola et al., 1999; Ayres et al., 2012). Therefore, the SC trials would be easy since there is a large difference in tastiness between the options, and energy content is relatively irrelevant. The NSC trials on the other hand would pose a hedonic dilemma for non-weight-concerned individuals since they were required to choose between two equally liked snacks. The finding, that our population of weight-concerned women perceived more conflict in the NSC trials in which the HE and LE foods were equal in tastiness and that in approximately half (54%) of these trials the LE snack was chosen suggests that choices were mainly based on tastiness considerations, and not influenced by energy content. The higher conflict in the NSC trials might therefore be interpreted as a hedonic dilemma of choosing between two equally liked snacks. Thus, intriguingly, both the choice patterns and the responses on the implicit measures in our population of self-proclaimed weight-concerned women resemble those expected for non-weight-concerned individuals. Because we used highly stringent inclusion criteria, it is likely that our study population truly had an authentic long-term goal to watch their weight. Therefore our results suggest that weight-concerned women's choices are made primarily on basis of taste considerations and not on basis of energy content. Thus, our results confirm other findings that suggest that self-reports of weight-concerns and restraint are reflective of intentions and wishes to restrict intake, rather than of actual eating behavior (Stice et al., 2004, 2007, 2010; de Witt Huberts et al., 2013; de Ridder et al., in press). It is of high interest to repeat this paradigm in a non-weight-concerned population. By comparing our results with a group of non-weight-concerned women, we could rule out whether the effects seen in the present study are general effects that occur also in non-weight-concerned women or whether they are specific for weight-concerned women in which the self-control dilemma is relevant.

Importantly, the present study is distinct from earlier neuroimaging studies on self-control because we focused specifically on conflict-monitoring in response to choice sets that either threaten the accomplishment of the long-term goal or not. Conflict monitoring is the process by which the brain determines when control is required (Botvinick et al., 2001) and thus precedes the actual act of self-control. Other neuroimaging studies on self-control have established how factors such as healthiness and tastiness are incorporated in the brain during food choices, how explicitly cueing people to consider healthiness of foods influences the neural response during food choice, and how depletion affects the neural response to rewarding food cues (Hare et al., 2009, 2011; Wagner et al., 2013).

It is important to note that we chose to use pairs of HE and LE snacks matched on tastiness as no self-control choices. From a theoretical perspective, choosing between equally liked HE and LE snacks should have constituted an easy choice for weight-concerned women: choosing LE snacks requires no self-control when they are equally tasty as the HE snack and also in line with their weight-watching goal. An alternative approach would have been to let participants choose between two LE foods differing in tastiness: this choice set also requires no self-control. However, contrasting choices between pairs of HE/LE snacks and pairs of LE/LE snacks has other disadvantages. For example, many earlier studies have shown that the neural response to HE and LE foods differs (van der Laan et al., 2011). Therefore, using pairs of LE snacks instead of our current NSC trials (pairs of HE and LE snacks), would have confounded our factor of interest—the need for self-control—with the energy content of the stimuli. For this reason, we chose to use pairs of equally liked HE and LE snacks as NSC trials.

Another factor which might have played a role is that tastiness ratings to match the choice pairs were collected prior to actual choice. It could be that LE snacks were rated as being tastier before the choice as compared to during choice, which is supported by an earlier study (Myrseth et al., 2009) that showed that activities in line with the long-term goal are rated higher in perceived appeal before making a decision than at the moment of (or after) deciding, when a more tempting alternative is concurrently available. However, it is unlikely that this would have affected the results for our contrast between NSC and SC trials because (1) the bias would be small (Myrseth et al., 2009), (2) the difference in tastiness between HE and LE snacks is still larger in the SC trials, posing a stronger conflict, than in the NSC trials, (3) using tastiness ratings collected prior to actual choices to match choice pairs on basis of pre-existing preference is an often used and well-acknowledged approach to study self-control (e.g., Hare et al., 2009), and (4) the finding that the proportion of HE and LE snacks in the NSC trials was approximately 50/50 suggests that choices were based on tastiness and that this tastiness was approximately equal.

Although the number of successful SC trials (SC trials in which the LE snack was chosen) was low, the eye tracking data revealed interesting patterns that hint toward a possibly effective strategy for successful self-control. The higher reaction times and number of gaze switches during successful compared to unsuccessful SC trials confirm the general assumption that it is difficult to choose a LE snack over a tastier HE alternative. The findings that participants fixated longer on LE than HE items in the successful SC trials and that the last fixation was significantly more often on the LE snack might have increased preference for the LE snack. Research has shown that fixation duration both reflects and influences preference. That is, people look longer at preferred or chosen items (Chandon et al., 2009; Atalay et al., 2012) and manipulating gaze duration for an option increases preference for it (Shimojo et al., 2003), i.e., there is a positive feedback loop between looking and liking. It could be speculated that directing attention away from preferred HE snacks and allocating attention to less-preferred LE snacks could break this loop and help people choose LE snacks. This idea is in line with the suggestion that distracting attention away from attractive stimuli might facilitate self-control for which Van Dillen et al. (2013) provided initial evidence. This topic deserves further investigation.

Our study population consisted of women with a healthy weight and therefore their motivation for weight-concern might not arise from medical or health reasons. Rather, since earlier studies showed a clear link between worries about appearance and weight-concerns/restraint (Putterman and Linden, 2004, 2006; O'Brien et al., 2007; de Ridder et al., in press) we think that the high level of self-reported weight-concerns in our study population might indicate an intention to lose weight for cosmetic reasons or general concerns about healthy eating. A large representative community study has also confirmed that high levels of restraint eating are mainly associated with concerns about weight and appearance and to a lesser extent associated with concerns about the perceived health consequences of dietary habits (de Ridder et al., in press). We would like to note that, although our population was of normal weight, research has shown that normal-weight individuals who report high weight-concerns are at increased risk for gaining weight (French et al., 1994; Mann et al., 2007). This is even worse for individuals that diet for appearance (as opposed to health) reasons: they show more lapses in restraint and disinhibited eating (Putterman and Linden, 2004). Therefore, normal weight females reporting high levels of weight-concerns are a very important population to focus on in research and weight-maintenance interventions, especially if you take into consideration that approximately 50–60% of the Western female population reports to be high in weight-concerns (Rideout and Barr, 2009; Fayet et al., 2012; de Ridder et al., in press).

We did not include self-reported measures of conflict in our study, that is, we did not ask them explicitly how difficult they found the choices or whether they consciously perceived conflict. Measuring subjective or self-reported measures of conflict in addition to implicit measures might have provided further understanding about consciously perceived conflict. However, we used implicit instead of subjective self-reported measures of conflict for several reasons. Firstly, the perception of conflict is not necessary a conscious phenomenon. Therefore, subjective measures might not validly capture the process. Secondly, implicit measures are less susceptible to demand characteristics and socially desirable responding (Fazio and Olson, 2003) than self-reported measures. Thirdly, ACC activity, reaction times, and fixation durations are well acknowledged measures to investigate response conflict (Kawashima et al., 1996; Macdonald et al., 2000; Botvinick et al., 2001; van Veen et al., 2001; Kerns et al., 2004; Panayiotou and Vrana, 2004; Causse et al., 2011). Finally, explicitly asking for response conflict during the food choices focusses attention on conflict which may also affect their choice.

Our findings have implications for weight management interventions. Since recognizing the self-control dilemma is a prerequisite for acting upon it, this study implies that weight management interventions might benefit from focusing on increasing the ability to detect a self-control dilemma, in addition to the current focus on increasing self-regulatory capacity and cognitive control. Evidently, if no conflict is experienced in the first place, improving the ability to perform effortful self-control is likely to be ineffective. As outlined above, a possible explanation for the lack of experienced conflict during food choice could be that the relevance of the long-term goal is over-ruled by the exposure to tempting foods. Therefore, a useful strategy might be to strengthen temptation-goal associations to change the automatic response to tempting foods to activating the long-term goal, rather than inhibiting it. Forming implementation intentions as an intervention has proven successful in strengthening temptation-goal associations (e.g., Kroese et al., 2011).

The lack of internal conflict in response to a food choice set posing a self-control dilemma may extrapolate to other behavioral domains involving a trade-off between direct impulses and future goals, such as smoking cessation, financial management, interpersonal relations and aggression. Future studies should elucidate whether a lack of experienced conflict also accounts for self-regulatory failure in these domains.

To conclude, in contrast with the common assumption that food choices posing a self-control dilemma evoke an internal conflict in weight-concerned women, we found that these choices appeared to induce no conflict, as indicated by lower reaction times, lower fixation durations, lower number of gaze switches between snacks, and decreased activation of the anterior cingulate cortex. These findings provide support for the notion that self-regulatory failure might be due to a lack of experienced conflict, rather than failing to act upon the experience of such conflict. Since recognizing the self-control conflict is a prerequisite for acting upon it, this study suggests that the effectiveness of weight maintenance interventions might be increased if they also focus on increasing the ability to detect a self-control dilemma, in addition to the current focus on increasing self-regulatory capacity and cognitive control.

## Author contributions

Laura N. Van der Laan, Paul A. M. Smeets, and Denise T. D. de Ridder conceived and designed the experiment. Laura N. Van der Laan collected and analyzed the data. Laura N. Van der Laan, Paul A. M. Smeets, and Denise T. D. de Ridder interpreted the data. Laura N. Van der Laan wrote the manuscript and Paul A. M. Smeets and Denise T. D. de Ridder provided critical revisions. Lisette Charbonnier and Max A. Viergever contributed to the final version of the manuscript by interpreting results, reviewing and critically revising text. Lisette Charbonnier provided the food pictures. All authors approved the final version for submission and agreed to be accountable to for all aspects of the work.

### Conflict of interest statement

The authors declare that the research was conducted in the absence of any commercial or financial relationships that could be construed as a potential conflict of interest.
