# Supplementary material for: Sweet lies: neural, visual, and behavioral measures reveal a lack of self-control conflict during food choice in weight-concerned women
Source: Front Behav Neurosci. 2014 May 22;8:184. doi: 10.3389/fnbeh.2014.00184 (PMC4033222; doi:10.3389/fnbeh.2014.00184)
Supplement: Supplementary file 1 [file DataSheet1.DOCX]

**Supplemental Tables**

**Table S1** Brain regions (whole brain results^1^) differentially activated for SC versus NSC trials.

|  |  |  | MNI coordinates | | | Cluster size |  |
| --- | --- | --- | --- | --- | --- | --- | --- |
| Cluster | Anatomical label | Side | x | y | z | voxels | Z value |
| *NSC minus SC trials* | |  |  |  |  |  |  |
| 1 | Frontal inferior gyrus, orbital part | L | -42 | 8 | 22 | 23 | 3.91 |
| 2 | Anterior cingulum | L | -10 | 28 | 26 | 63 | 3.51 |
|  | Frontal superior gyrus, medial part | L | -6 | 28 | 42 |  | 3.31 |
|  | Anterior cingulum | R | 10 | 28 | 30 |  | 3.18 |
| 3 | Inferior frontal gyrus, orbital part | L | -42 | 20 | -2 | 38 | 3.44 |
|  | Insula | L | -30 | 24 | -6 |  | 2.99 |
| 4 | Insula | R | 34 | 28 | -2 | 20 | 3.31 |
| 5 | Calcarine gyrus | R | -6 | -92 | -2 | 12 | 2.95 |
| *SC minus NSC trials* | |  |  |  |  |  |  |
| 1 | Supramarginal gyrus | R | 62 | -28 | 46 | 122 | 3.83 |
|  | Postcentral gyrus | R | 26 | -40 | 66 |  | 3.09 |
|  | Precuneus | L | -10 | -52 | 58 |  | 3.07 |
| 2 | Superior frontal gyrus | R | 22 | 4 | 50 | 41 | 3.50 |
|  | Supplementary motor area | R | 14 | 4 | 58 |  | 3.00 |
| 3 | Middle temporal gyrus | R | 66 | -44 | 2 | 29 | 3.01 |
|  | Middle temporal gyrus | R | 70 | -36 | 2 |  | 3.01 |

^1^ Peaks of clusters significant at p < 0.005 and k ≥ 10 voxels are reported

**Table S2** Multi-level regression results for reaction time in the food choice task. Independent variables: trial category (SC or NSC). Dependent variable: log-transformed reaction time.

| Model effect | Estimate | Std. Error | t | p |
| --- | --- | --- | --- | --- |
| *Fixed effects* |  |  |  |  |
| Intercept | 1.45 | 0.06 | 25.84 | <0.01 |
| Trial category: SC | -0.10 | 0.02 | -4.60 | <0.01 |
|  |  |  |  |  |
| *Random effects* | Variance | SD |  |  |
| Intercept (level 2 participant) | 0.16 | 0.40 |  |  |
|  |  |  |  |  |
| Log likelihood model | -769.6 |  |  |  |
| AIC | 1547 |  |  |  |

**Table S3** Multi-level regression results for total fixation duration. Independent variables: trial category (SC or NSC). Dependent variable: total fixation duration per trial (sum of left and right snack).

| Model effect | Estimate | Std. Error | t | p |
| --- | --- | --- | --- | --- |
| *Fixed effects* |  |  |  |  |
| Intercept | 0.97 | 0.06 | 17.18 | <0.01 |
| Trial category: SC | -0.11 | 0.02 | -5.17 | <0.01 |
|  |  |  |  |  |
| *Random effects* | Variance | SD |  |  |
| Intercept (level 2 participant) | 0.05 | 0.22 |  |  |
|  |  |  |  |  |
| Log likelihood model | -721.6 |  |  |  |
| AIC | 1451 |  |  |  |

**Table S4** Multi-level regression results for lexical decision task measuring temptation-goal associations. Independent variables: target word (neutral or diet), prime word (neutral or temptation), and interaction between target word en prime word. Dependent variable: (log) reaction time.

| Model effect | Estimate | Std. Error | t | p |
| --- | --- | --- | --- | --- |
| *Fixed effects* |  |  |  |  |
| Intercept | 6.25 | 0.03 | 200.29 | <0.01 |
| Prime word | -0.01 | 0.01 | -0.48 | 0.63 |
| Target word | -0.01 | 0.01 | -0.47 | 0.64 |
| Interaction prime X target | 0.00 | 0.02 | 0.22 | 0.83 |
|  |  |  |  |  |
| *Random effects* | Variance | SD |  |  |
| Intercept (level 2 participant) | 0.02 | 0.13 |  |  |
|  |  |  |  |  |
| Log likelihood model | 85.72 |  |  |  |
| AIC | -159.4 |  |  |  |
